# A Common Food Glycan, Pectin, Shares an Antigen with Streptococcus pneumoniae Capsule

**DOI:** 10.1128/mSphere.00074-20

**Published:** 2020-04-08

**Authors:** Moon H. Nahm, Jigui Yu, Jiri Vlach, Maor Bar-Peled

**Affiliations:** a Department of Medicine, Division of Pulmonary, Allergy & Critical Care, University of Alabama at Birmingham, Birmingham, Alabama, USA; b Department of Microbiology, University of Alabama at Birmingham, Birmingham, Alabama, USA; c Complex Carbohydrate Research Center, University of Georgia, Athens, Georgia, USA; U.S. Food and Drug Administration

**Keywords:** food, bacteria, vaccine, polysaccharide, *Streptococcus pneumoniae*, capsule

## Abstract

We are exposed daily to many glycans from bacteria and food plants. Bacterial glycans are generally antigenic and elicit antibody responses. It is unclear if food glycans’ sharing of antigens with bacterial glycans influences our immune responses to bacteria. We studied 14 different plant foods for cross-reactivity with monoclonal antibodies (MAbs) against 24 pneumococcal serotypes which commonly cause infections and are included in pneumococcal vaccines. Serotype 15B-specific MAb cross-reacts with fruit peels, and serotype 10A MAb cross-reacts with many natural and processed plant foods. The serotype 10A cross-reactive epitope is 1,6-β-galactosidase [βGal(1-6)], present in the rhamno-galacturonan I (RG-I) domain of pectin. Despite wide consumption of pectin, the immune response to 10A is comparable to the responses to other serotypes. An antipectin antibody can opsonize serotype 10A pneumococci, and the shared βGal(1-6) may be useful as a simple vaccine against 10A. Impact of food glycans should be considered in host-pathogen interactions and future vaccine designs.

**IMPORTANCE** The impact of food consumption on vaccine responses is unknown. Streptococcus pneumoniae (the pneumococcus) is an important human pathogen, and its polysaccharide capsule is used as a vaccine. We show that capsule type 10A in a pneumococcal vaccine shares an antigenic epitope, βGal(1-6), with pectin, which is in many plant foods and is widely consumed. Immune response to 10A is comparable to that seen with other capsule types, and pectin ingestion may have little impact on vaccine responses. However, antibody to pectin can kill serotype 10A pneumococci and this shared epitope may be considered in pneumococcal vaccine designs.

## Abstract (precis)

The impact of food consumption on vaccine responses is unknown. Streptococcus pneumoniae (the pneumococcus) is an important human pathogen, and its polysaccharide capsule is used as a vaccine. We show that capsule type 10A in a pneumococcal vaccine shares an antigenic epitope, βGal(1-6), with pectin, which is in many plant foods and is widely consumed. Immune response to 10A is comparable to that seen with other capsule types, and pectin ingestion may have little impact on vaccine responses. However, antibody to pectin can kill serotype 10A pneumococci and this shared epitope may be considered in pneumococcal vaccine designs.

## INTRODUCTION

The human body is constantly exposed to a large array of foreign glycans (carbohydrate-containing polymers such as polysaccharides [PS], glycolipids, and glycoproteins). One source of foreign glycans is represented by the bacteria that live around us as well as within us. Bacterial glycans include teichoic acid and peptidoglycan made as a part of bacterial cell walls. Among the other glycans are many different types of capsules, lipopolysaccharides (LPS), and exopolysaccharides. For instance, salmonella and Streptococcus pneumoniae (the pneumococcus), two well-known human pathogen species, can produce about 50 different LPS structures (1) and 100 different capsule types (2), respectively, all differing in sugar composition and/or linkages. The pneumococcal capsule is a major virulence factor and is successfully used in vaccines since anticapsule antibodies (Abs) are highly protective. Pneumococcal teichoic acid and capsular polysaccharides are also secreted into urine, allowing diagnostic tests of urine to be used to detect pneumococcal infections (3, 4).

Food from plants represents another source of foreign glycan exposure. Plants produce myriads of glycans to store energy and synthesize structural components. Starch is a typical energy storage glycan, and cell wall polysaccharides provide plants with structure. The cell wall glycans include cellulose, hemicellulose, and pectin (5). Pectin itself is a structurally complex polysaccharide (6) that includes homogalacturonan (∼65%), rhamno-galacturonan I (RG-I) (∼20 to 35%), and rhamno-galacturonan II (RG-II) (∼10%) (6). Humans regularly ingest pectin since it is a component of fruits, vegetables, and processed foods such as jams.

Since plant and bacterial glycans are diverse, some of them may be antigenically similar. If antigenic similarity exists, ingesting food containing cross-reactive glycans may elicit antibodies to bacterial glycans or influence bacterial vaccine responses or diagnostic tests. It is even possible that our immune system may undergo tolerization and may not respond to bacterial glycans cross-reacting with common food items. To examine these possibilities, we have examined several glycan-containing food items for antigens cross-reactive with pneumococcal capsules.

## RESULTS

### Fruits and vegetable extracts contain materials that cross-react with capsular polysaccharide of pneumococcal serotypes 10A and 15B.

To investigate if food from plants can share epitopes with pneumococcal capsules, we obtained 14 different food items from a grocery store and tested their extracts (4% [wt/wt]) for cross-reaction in our bead array assay with 26 pneumococcal capsule-specific monoclonal antibodies (MAbs) (against serotypes 1, 2, 3, 4, 5, 6A, 6B, 6C, 6D, 7F/7A, 8, 9N, 9V, 10A, 11A, 12F, 14, 15B, 17F/17A, 18C, 19A, 19F, 20, 22F/22A, 23F, and 33F/33A) (7). Except for serotypes 6C and 6D, all of these serotypes are included in one or more pneumococcal vaccines (2). All 14 plant extracts cross-reacted with the 10A antibody, with titers ranging from 16 for cucumber to 4,380 for carrots (Table 1). In addition, three extracts (orange, orange peel, and tangerine peel) showed some reactivity with the 15B monoclonal antibody (Table 1). No food items showed demonstrable cross-reactivity with antibodies for any of the other serotypes (data not shown).

**TABLE 1:** Cross-reactive material in fruits and vegetables^*a*^

Fruit or vegetable category	10A titer	15B titer
Apples	37	<10
Bell pepper	63	<10
Broccoli	51	<10
Carrots	4,380	<10
Cauliflower	470	<10
Cantaloupe	46	<10
Celery	517	<10
Cucumber	16	<10
Okra	23	<10
Orange	922	∼10
Orange peel	631	72
Spinach	39	<10
Tangerine peel	575	∼10
Tomato	72	<10

a Each titer value indicates the sample dilution that inhibits 50% of binding.

### RG-I pectin cross-reacts with serotype 10A.

**FIG 1:**
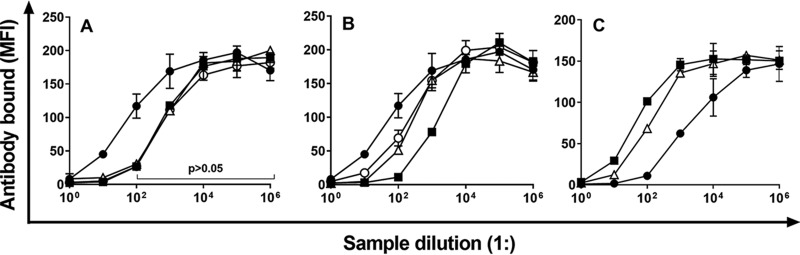
(A) Amount of monoclonal antibody bound to microbeads coated with serotype 10A polysaccharide in the presence of various concentrations of 10A polysaccharide (filled circle), raw carrot extract (filled square), acid-treated carrot extract (open circle), and base-treated carrot extract (open triangle). As shown in the panel, both acid and alkali hydrolysates are comparable to the raw extract in their levels of inhibition at dilutions greater than 100-fold (*P > *0.05). Initial concentrations were 10 μg/ml for 10A polysaccharide and neat for the carrot extract. Error bars indicate standard deviations (SDs) and are not visible where SDs are smaller than the symbol itself. (B) Amount of monoclonal antibody bound to 10A-coated microbeads in the presence of serial dilutions of 10A polysaccharide (filled circle), strawberry jam (open triangle), pureed carrot for infants (filled square), and apple juice (open circle). Initial concentrations were 10 μg/ml for 10A polysaccharide and a 1:4 dilution for the jam and pureed carrot. Error bars indicate SDs and are not visible where SDs are smaller than the symbol itself. (C) Amount of monoclonal antibody bound to 10A-coated microbeads in the presence of 10A polysaccharide (filled circle), apple pectin (filled square), and pectin from citrus fruits (open triangle) as inhibitors. The initial concentration of the inhibitors was 500 μg/ml. The monoclonal antibody was Hyp10AG1. Error bars indicate SDs and are not visible where SDs are smaller than the symbol itself. MFI, mean fluorescence intensity.

Since the 10A cross-reactivity was consistent and strong, we further investigated the nature of the 10A cross-reactive material in the carrot. The cross-reactivity of the carrot material was retained even after incubation with an acid (0.2 M HCl, 2 h at 37°C) or a base (0.2 M NaOH, 2 h at 37°C) (Fig. 1A). The cross-reactive material was also found in processed foods such as a baby food (pureed carrot), apple juice, and strawberry jam (Fig. 1B).

Pectin is the gelling material for jams and is resistant to acid and base (6). Thus, commercially available pectin from apple or citrus fruits was tested for cross-reactivity with a monoclonal antibody specific for serotype 10A (Hyp10AG1). Both pectins bound unambiguously to a 10A-specific monoclonal antibody, although the pectins showed about 30-fold to 70-fold less cross-reactivity than 10A polysaccharide by weight (Fig. 1C). Similar cross-reactivity was observed with another 10A monoclonal antibody (Hyp10AM6) (see Fig. S1 in the supplemental material).

10.1128/mSphere.00074-20.1 FIG S1 Hyp10AM6 also binds to pectin. Data represent the amount of monoclonal antibody bound to 10A-coated microbeads in the presence of 10A polysaccharide (filled triangle), apple pectin (star), and pectin from citrus fruits (filled square) as inhibitors. The monoclonal antibody was Hyp10AM6. Download FIG S1, TIF file, 1.0 MB. Copyright © 2020 Nahm et al. 2020 Nahm et al. This content is distributed under the terms of the Creative Commons Attribution 4.0 International license.

**FIG 2:**
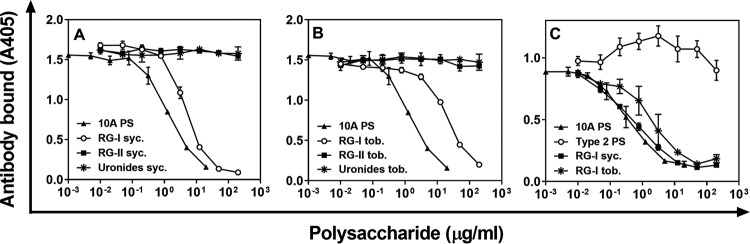
(A and B) Amount of Hyp10AG1 bound to 10A-coated ELISA wells in the presence of serotype 10A capsular polysaccharide (filled triangle), RG-I (open circle), RG-II (filled square), or homogalacturonan (star, indicated as uronides). Pectin was from sycamore (syc.) (A) or from tobacco (tob.) (B). (C) Amount of CCRC-M7 bound to 10A-coated ELISA wells in the presence of 10A polysaccharide (filled triangle), serotype 2 polysaccharide (open circle), and RG-I pectins from sycamore (filled square) and tobacco (star). Error bars indicate SDs and are not visible where SDs are smaller than the symbol itself.

Pectin has three major structural components: RG-I, RG-II, and homogalacturonan (6). We investigated the cross-reactivity with pectin components purified from two model species, sycamore and tobacco. Sycamore RG-II and small homogalacturonans showed no detectable cross-reaction with Hyp10AG1 (Fig. 2A). However, sycamore RG-I showed cross-reactivity with Hyp10AG1 that was 3-fold lower than the cross-reactivity exhibited by 10A polysaccharide (Fig. 2A). Similar observations were made with tobacco pectin components, but with tobacco RG-I being about 50-fold less cross-reactive than 10A polysaccharide (Fig. 2B). RG-I pectin from Arabidopsis, another model species, was also cross-reactive (Fig. S2). In contrast, a monoclonal antibody to RG-I (CCRC-M7) (8) reacted with 10A polysaccharide as well as with sycamore and tobacco RG-I (Fig. 2C). This monoclonal antibody (MAb) was produced by immunizing mice with sycamore RG-1 (8) and is available from the Complex Carbohydrate Research Center of the University of Georgia in Athens, GA. Thus, the RG-I component of pectin has an epitope that cross-reacts with various 10A-specific monoclonal antibodies.

10.1128/mSphere.00074-20.2 FIG S2 RG-I pectin from Arabidopsis binds a 10A-specific antibody. Data represent the amount of Hyp10AG1 bound to 10A-coated ELISA wells in the presence of serotype 10A capsular polysaccharide (filled triangle), RG-I from Arabidopsis (open circle), pectin from citrus fruits (filled square), apple pectin (star), and RG-II from wine (solid diamond). Download FIG S2, TIF file, 0.9 MB. Copyright © 2020 Nahm et al. 2020 Nahm et al. This content is distributed under the terms of the Creative Commons Attribution 4.0 International license.

### βGal(1-6) is the shared epitope between RG-I and 10A polysaccharide.

**FIG 3:**
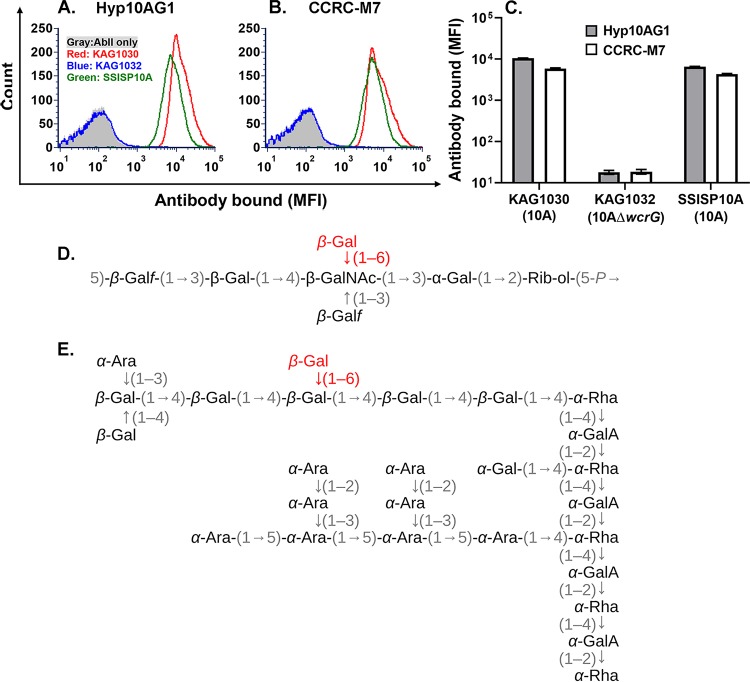
(A and B) Binding of a MAb against pneumococcal capsule type 10A (Hyp10AG1) (A) and against pectin (CCRC-M7) (B) as determined by flow cytometry to pneumococci with intact *wcrG* (SSISP10A and KAG1030) or defective *wcrG* (KAG1032, 10AΔ*wcrG*). Gray peak shows binding without the MAbs. (C) Amount of Hyp10AG1 and CCRC-M7 bound to strain KAG1030, KAG1032, or SSISP10A. Error bars show SD, and binding of both MAbs to 10AΔ*wcrG* (KAG1032) was significantly reduced compared to the level seen with 10A (KAG1030 or SSISP10A) (*P* < 0.001). (D) Structure of 10A capsular polysaccharide (2). (E) Diagram of a fragment of rhamno-galacturonan-I (RG-I) pectin molecule. (Adapted from reference 35.) Rhamno-galacturonan backbone of pectin is shown to the right. The βGal(1-6) shared between 10A polysaccharide and RG-I is bolded and red. Gal = d-galactopyranosyl; GalA = d-galacturonosyl; Rha = l-rhamnopyranosyl; Ara = l-arabinofuranosyl; Gal*f* = d-galactofuranosyl; GalNAc = 2-acetamido-2-deoxy-d-galactosyl; Rib-ol = d-ribitol.

Both RG-I and 10A polysaccharide have terminal 1,6-β-galactosidase [βGal(1-6)] (Fig. 3), and a 10A-specific pneumococcal antibody recognizes this epitope on 10A polysaccharide (9). To directly show that βGal(1-6) is the epitope shared between RG-I and 10A polysaccharide, we studied two 10A pneumococcal strains (KAG1030 and SSISP10A) and strain KAG1032, a 10A variant lacking *wcrG*. *wcrG* encodes the galactosyltransferase responsible for the terminal βGal(1-6) (9), and KAG1032 was created from KAG1030 by replacing *wcrG* with a kanamycin resistance gene. When the bacterial strains were examined with the two monoclonal antibodies (Hyp10AG1 and CCRC-M7), both monoclonal antibodies bound to strains with intact *wcrG* (SSISP10A and KAG1030) but not to the *wcrG*-deficient strain (KAG1032) (Fig. 3). Similarly, the two monoclonal antibodies did not bind a serotype 10B strain, which produces capsule similar to that produced by the serotype 10A strain but without βGal(1-6) (data not shown). Thus, we conclude that (1-6)-linked β-d-Gal is the epitope shared by 10A polysaccharide and RG-I.

### Biological impact of pectin and pectin antibody.

**FIG 4:**
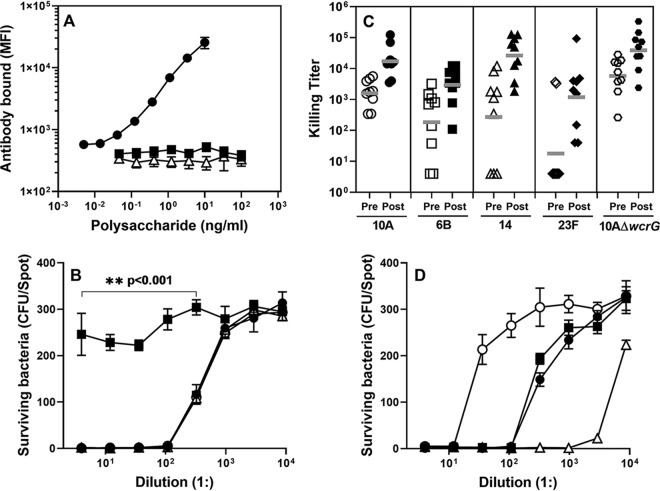
(A) Amount of rabbit antibody bound to the microspheres coated with a monoclonal antibody against 10A polysaccharide (Hyp10AM6) in the presence of various polysaccharide samples. Error bars indicate SDs and are not visible where SDs are smaller than the symbol itself. Rabbit antibodies bound significantly more in the presence of only 0.12 ng/ml of 10A polysaccharide (solid circle) than in the presence of even 100 ng/ml of sycamore RG-I (solid square) or tobacco RG-I (open triangle) (*P < *0.005). (B) Surviving pneumococci (*y* axis) following opsonophagocytic killing assay at various dilutions of 007sp (*x* axis) in the presence of inhibitors. Inhibitors were as follows: none (buffer only, solid circle), 10 μg/ml of 10A polysaccharide (solid square), 100 μg/ml of type 2 capsular polysaccharide (open triangle), and 500 μg/ml of RG-I (open circle). 007sp was prediluted 20-fold. As indicated in the panel, control polysaccharides differed significantly from 10A polysaccharide in preventing the killing (*P < *0.001). At 100-fold dilution, almost no bacteria survived in the presence of control polysaccharide but almost all bacteria survived with 10A polysaccharide (*P < *0.001). (C) The opsonophagocytic killing titers of preimmune (open symbols) and postimmune (filled symbols) sera from nine individual donors. The target serotypes are shown at the bottom, and the horizontal gray bars in the figure indicate geometric mean values. Killing titers significantly increased for all serotypes following vaccination (*P* = 0.00002 for serotype 10A, 0.0056 for 6B, 0.00045 for 14, 0.0057 for 23F, and 0.00026 for 10AΔ*wcrG*). Pre, preimmune; Post, postimmune. (D) Surviving pneumococci (*y* axis) following opsonophagocytic killing assay at various dilutions of antibodies (*x* axis). The antibodies were 007sp (filled circle), Hyp10AG1 (filled square), Hyp10AM6 (open triangle), and CCRC-M7 (open circle). Hyp10AG1, Hyp10AM6, and CCRC-M7 culture supernatants were prediluted 2-fold before the assay. 007sp, a reference human serum for pneumococcal assays (36), was prediluted 20-fold. Error bars indicate SDs and are not visible where SDs are smaller than the symbol itself.

Since pectin is consumed in large amounts, small amounts may be absorbed and produce various forms of immunological interference. To investigate this possibility, we first investigated if pectin could influence a urinary capsule test (UCT). The UCT is an immunoassay for pneumococcal capsule in urine and is used to identify serotypes causing nonbacteremic pneumonia (4). The UCT that we used for this investigation is a sandwich-type immunoassay using 10A-specific monoclonal antibody (Hyp10AM6) and a rabbit anti-capsule immune serum (Staten Serum Institute). This UCT was able to detect less than 1 ng/ml of 10A polysaccharide in urine samples but was not able to detect even 100 ng/ml of sycamore or tobacco RG-I (Fig. 4A). Also, within the first 24 h after ingestion of carrots, no urine specimens were obtained that contained any material that would interfere with the 10A UCT (data not shown). Further, 100 ng/ml of RG-I was unable to inhibit 3 to 10 ng/ml of 10A polysaccharide from being detected by our UCT (data not shown), suggesting that a 30-fold excess amount of pectin was unable to displace 10A polysaccharide. Thus, RG-I pectin binds to 10A-specific antibodies so weakly that ingestion of large amounts of pectin would be unlikely to interfere with a UCT.

Next, we determined if absorbed pectin could inhibit the functionality of antibodies to 10A polysaccharide in blood by determining if RG-I pectin could inhibit the opsonic capacity of a human serum pool (007sp) containing 10A antibodies. 007sp is a pool of sera from adults immunized with a pneumococcal polysaccharide vaccine (10), and the *in vitro* opsonization assay is widely used for testing pneumococcal vaccines (11). As expected, 10 μg/ml of 10A polysaccharide completely inhibited the opsonic capacity of 007sp and an unrelated capsular polysaccharide (serotype 2) did not inhibit its opsonic capacity (Fig. 4B). RG-I did not inhibit the human serum pool at a high (500 μg/ml) concentration (Fig. 4B). A similar observation was made with a rabbit antiserum against 10A (factor serum 10d) instead of 007sp (data not shown). Thus, even if pectin were present at a high concentration in blood, the pectin would not reduce the functional capacity of human antibodies to 10A.

Serotype 10A pneumococci often cause pneumococcal infections, with 10A polysaccharide being included in the 23-valent polysaccharide vaccine. We examined nine vaccinees immunized with this vaccine for their immune responses to serotype 10A as well as to three other serotypes in this vaccine and a 10A strain with defective *wcrG* (KAG1032, 10AΔ*wcrG*). Prevaccination titers of antibodies were low for all the five serotypes, including 10A and 10AΔ*wcrG*, but vaccination resulted in large and comparable increases in antibody titers to all serotypes, including 10A and 10AΔ*wcrG* (Fig. 4C). This finding suggests that most anti-10A antibodies target the framework of 10A polysaccharide. Also, even though pectin is ubiquitous in our diet, exposure to pectin appears to have little influence on either the prevaccination level of or the immune responses to 10A polysaccharide.

In view of the data presented above, we determined if a pectin antibody targeting the βGal(1-6) could opsonize serotype 10A pneumococci at all. We found that a pectin-specific monoclonal antibody did opsonize and kill pneumococci, the same as monoclonal antibodies against 10A polysaccharide, in an *in vitro* opsonization assay (Fig. 4D). Further, the opsonization was specific to 10A as the monoclonal antibody did not opsonize pneumococci expressing irrelevant serotypes (data not shown). Thus, pectin antibodies can opsonize pneumococci expressing serotype 10A even though the cross-reaction may not be strong enough to efficiently block the interaction between 10A-specific antibodies and 10A polysaccharide.

## DISCUSSION

Although many studies have found that bacterial polysaccharides can resemble animal glycans (i.e., antigen mimicry) (12, 13), there have been very few studies comparing bacterial glycans with the plant glycans we eat (14, 15) and no studies performed at the molecular level. We now provide a new insight by showing unambiguously that RG-I pectin from plants and food items shares epitopes with pneumococcal capsule type 10A and that antipectin antibody can kill 10A pneumococci. While pectin cross-reacts with anti-10A antibodies, the antibodies preferentially react with 10A and low concentrations of pectin cannot inhibit interactions between 10A and anti-10A antibodies. The shared epitope is the terminal (nonreducing) βGal(1-6) in their side chains, since removal of the βGal(1-6) side chain from 10A polysaccharide eliminates its reaction with a monoclonal antibody to 10A polysaccharide (Hyp10AG1) and with a monoclonal antibody to RG-I (CCRC-M7) (8).

We also discovered another antigen shared between serotype 15B and fruit peels. Since the 15B-specific monoclonal antibody recognizes the O-acetylated αGal(1-2) side chain (16), we predicted that an analogous structure would be present in fruit peels. We did not detect a cross-reaction between okra and serotype 6A polysaccharide where we tested only one 6A-specific monoclonal antibody. However, it is possible that they still may share epitopes as suggested in the past (14). Of significance is that we have discovered two shared antigens by studying only 14 different foods from plants and 26 bacterial glycans. Given that plants produce myriads of polysaccharides and that each glycan would express multiple antigenic sites, there should be many more glycan antigens shared among bacteria and plant-based foods.

If the shared glycan antigens are present in common food items, these antigens would accompany our body since birth and could influence human immune responses or host defense. An example of such a “companion antigen” is αGal(1-3)-βGal(1-4)-GlcNAc present in red meat. This antigen is produced by a galactosyltransferase (GGTA1) that is present in animals but not in humans (17). A 1% concentration of human IgG targets the antigen (17); thus, the antibody may be protective against the normal intestinal flora expressing LPS with the antigen (18). IgE antibodies are also created in some individuals following tick bites and cause an allergy to red meat (19, 20). Another example of a food antigen that interferes with immune responses in humans is the N-glycolylneuraminic acid present in red meat. Its ingestion creates the target for subtilase cytotoxin secreted by a pathogenic Escherichia coli strain (21) and may trigger chronic inflammation, termed “xenosialitis” (22).

Like red meat, pectin is widely consumed, with its βGal(1-6) being another companion antigen. So far, we have found little evidence for pectin influencing our immune response to pneumococci. The vaccine response to the 10A serotype is as robust as the response to other serotypes, and the prevaccination level of anti-10A is low. Further, for the reasons discussed below, pectin may have little impact on the functioning of preexisting pneumococcal antibodies. First, although one may consume up to 5 to 20 gm of pectin per day (23), pectin is a soluble fiber that is poorly absorbed. Indeed, we failed to detect evidence of the presence of 10A-like material in urine of persons who ingested carrots. However, it is possible that pectin may be absorbed and cause analytical interference in some persons with intestinal disorders such as inflammatory bowel diseases. Second, RG-I pectin binds to anti-10A antibodies more weakly than does 10A polysaccharide itself. Consequently, even if a small amount of pectin is absorbed, the amount of absorbed pectin should not be high enough to inhibit the production of endogenous anti-10A antibodies. As our tests showed, a relatively high level of pectin (500 μg/ml) did not inhibit the opsonic capacity of anti-10A antibodies and pectin did not interfere with a sensitive diagnostic assay for 10A polysaccharide in urine.

An unexpected observation from our results is that a pectin-specific monoclonal antibody opsonizes serotype 10A pneumococci for phagocytes. Pectin has very little similarity in chemical structure with 10A polysaccharide except for the βGal(1-6) branch. Also, the terminal βGal(1-6) is known to be immunogenic in multiple animal species: βGal(1-6) is targeted by mouse monoclonal antibodies (9) and by a rabbit antiserum used for pneumococcus typing (factor serum 10d) (24). Simple structures such as terminal βGal(1-6) or βGal(1-6)-βGalNAc may then function as a pneumococcal vaccine against 10A serotype. Since these simple structures can be readily synthesized by chemical means, one should be able to significantly simplify pneumococcal conjugate vaccine designs.

Plant materials expressing vaccine antigens have been investigated as “edible vaccines” (25). Although vaccines against polio, rotavirus, or cholera can be given orally and induce protective responses (26), edible vaccines are still in early stages of development (27). A barrier that has yet to be overcome is the identification of factors controlling the induction or suppression of immune responses (28) following oral ingestion of an antigen, as seen with development of either allergy or tolerization following a food intake (29). Pectin naturally expresses a pneumococcal vaccine antigen and is readily available in both natural foods and as a purified material. Thus, pectin would be useful in edible vaccine development by helping one identify factors enhancing or reducing the immunogenicity of edible vaccines.

## MATERIALS AND METHODS

### Preparation of vegetable and fruit extracts.

Fourteen different species of fruits and vegetables, apple juice, strawberry jam (Smucker’s), and pureed carrot (Beech-Nut baby food) were obtained from a local grocery store. Crude water-soluble extracts of the plant’s edible parts were prepared by mixing 1 part (by weight) of plant material with 4 parts (by weight) of distilled water, blending the mixtures at a high-speed setting in a Windmere model B55 blender for 2 min, and leaving the mixtures at 4°C overnight. Extracts from strawberry jam and pureed carrot were prepared by diluting them 1:4 with water, mixing them vigorously, and then leaving them at 4°C overnight. The next day, supernatants from all extracts were separated from insoluble matter by centrifugation (Sorvall RT6000; ∼1,000 × g).

### Purification of pectin.

RG-I, RG-II, and homogalacturonan were prepared as described previously (30, 31) by digestion of cell wall obtained from tobacco or sycamore with endopolygalacturonases (EPGs). The resulting partially digested pectin molecules in the supernatant were dried, solubilized in 50 mM NaOAc (pH 5.2), and separated over a P30-Biogel column or Sephadex G-75 column preequilibrated with sodium acetate. Pectic polymer fractions were also obtained from wine and Arabidopsis EPG-treated cell walls. For example, a typical preparation of pectic polymers from 10 g of Arabidopsis walls yielded 85 mg RG-I, 22 mg RG-II, and 15 mg homogalacturonans.

### Inhibition multiplex assay for pneumococcal capsule types.

The assay was performed as described before (7). Twenty-five microliters of 10A polysaccharide-coated microbead suspension was mixed with 25 μl of diluted polysaccharide standard or pectin and 25 μl of a 10A-specific monoclonal antibody (either Hyp10AG1 or Hyp10AM6) in each microtiter well. After a 30-min incubation at room temperature (RT), the beads in the microtiter plate were washed with the wash buffer. A 50-μl volume of phycoerythrin (PE)-conjugated goat anti-mouse immunoglobulin (BD Pharmingen, Franklin Lakes, NJ), which was diluted 1:200 in blocking buffer, was added to each well. The plates were incubated for 30 min with shaking. After washing was performed, the beads were resuspended in 125 μl of wash buffer and their fluorescence was determined with a flow cytometer (Bio-Pex 200; Bio-Rad Laboratories, Hercules, CA).

### Inhibition ELISA.

An inhibition-type enzyme-linked immunosorbent assay (ELISA) was performed as described previously (32). Briefly, the wells of ELISA plates were coated with 100 μl of 5 μg/ml of 10A capsular polysaccharide (ATCC, Manassas, VA)–phosphate-buffered saline (PBS) for 5 h of incubation at 37°C. After washing of the plates with PBS containing 0.05% Tween 20, serially diluted pectin or polysaccharide standards were added to the wells along with anti-10A (Hyp10AG1) or anti-pectin (CCRC-M7) monoclonal antibody. Apple and citrus pectins were from Sigma-Aldrich (St. Louis, Mo). CCRC-M7 was purchased from the Complex Carbohydrate Research Center (Athens, GA) and used at a 1:20 dilution, and Hyp10AG1 was used at a 1:30 dilution. After 1 h of incubation at 37°C, the plates were washed and incubated for 1 h with alkaline phosphatase-conjugated goat anti-mouse IgG (SouthernBiotech. Birmingham, AL). The amount of the enzyme immobilized in the wells was determined with *p*-nitrophenyl phosphate substrate–diethanolamine buffer. The optical density at 405 nm was read with a microplate reader (BioTek Instruments Inc., Winooski, VT).

### Sandwich-type assay for 10A polysaccharide.

One microliter of anti-10A antibody-coated bead suspension was added to each well of a 96-well plate, and the plate was then washed twice with wash buffer (PBS, 0.1% Tween 20, 0.02% sodium azide). The microbeads (Luminex Corporation, Austin, Texas) were coupled with a 10A-specific MAb (Hyp10AM6) as recommended by the manufacturer using [1-ethyl-3-(3-dimethylaminopropyl] carbodiimide and N-hydroxysulfosuccinimide (https://cdn2.hubspot.net/hubfs/128032/Cookbook/BR76862.xMAPCookbook.Ed4.WR.pdf; accessed 28 October 2015). A 50-μl volume of sample (polysaccharide standard, pectin, urine sample, or blocking buffer) was added to each well, and the plate was then incubated for 2 h at RT with shaking. Urine samples used for this study were obtained from individuals at 0, 1, 2, 6, and 24 h after ingestion of two carrots under an Institutional Review Board (IRB) approval (IRB-300002614). After washing of the beads was performed, 50 μl of a rabbit serum pool was added to each well and the plate was then incubated for 1 h at RT with shaking. The serum pool was made by mixing rabbit serum pools P, Q, R, S, T, E, and F from SSI Diagnostica (Copenhagen, Denmark) and a serum sample from a rabbit immunized with PCV13 (a gift from Mary Marquart, University of Mississippi Medical Center, Jackson MS) in equal volumes. After washing of the beads was performed twice, the beads were mixed with 50 μl of PE-goat anti-rabbit Ig (Southern Biotech, Birmingham, AL) and incubated for 30 min at RT with shaking. After washing was performed, the beads were resuspended in 130 μl of wash buffer, and their fluorescence was determined with a BD Accuri C6 plus flow cytometer (BD Biosciences, San Jose, CA).

### Genetic manipulations.

SSISP10A was made streptomycin resistant by transforming it with the mutant *rpsL* allele from TIGRJS (33). The resulting streptomycin-resistant transformant was named KAG1030. *wcrG* was deleted from the KAG1030 strain via allelic exchange with a Janus cassette, replacing *wcrG* with a kanamycin resistance gene (34). A kanamycin-resistant transformant (KAG1032) was selected after transformation of KAG1030 with this construct. Sequencing of *cps* loci of KAG1032 confirmed that the genetic manipulation had been performed correctly (data not shown).

### Flow-cytometric analysis.

Flow-cytometric analysis was performed with bacteria as described previously (33). Briefly, bacteria were incubated with a monoclonal antibody for 30 min at 4°C. After washing was performed, the bacteria were incubated with phycoerythrin-labeled anti-mouse immunoglobulin antibody (catalog no. 1030-09; Southern Biotech, Birmingham, AL). Flow cytometry data were obtained using a BD Accuri C6 plus flow cytometer (BD Biosciences, San Jose, CA) and analyzed using FCS Express (De Novo Software, Los Angeles, CA).

### Opsonophagocytosis assay.

Opsonophagocytosis assay was performed using the 4-fold multiplexed opsonization assay (11). Briefly, 10 μl of bacterial suspension and 20 μl of serially diluted anonymized human antiserum or a hybridoma supernatant were incubated in a microtiter plate for 30 min at RT. Serum samples were obtained from adults before and 1 month after immunization with a 23-valent pneumococcal polysaccharide vaccine. The reference serum used (007sp) has been described previously (10). The five target bacteria strains were OREP10A, SPEC6B, STREP14, EMC23F, and KAG1032, which represented serotypes 10A, 6B, 14, 23F, and 10AΔ*wcrG*, respectively.

The urine samples used for this study were obtained from individuals at 0, 1, 2, 6, and 24 h after ingestion of two carrots (about 200 gm) under the approval (IRB-300002614) of an IRB at the University of Alabama at Birmingham. The serum samples used for opsonophagocytosis assay were obtained from volunteers, who were immunized with a 23-valent pneumococcal polysaccharide vaccine under the approval (RSRB 07186) of an IRB in the University of Rochester (Rochester, NY).

## References

[1] Liu B, Knirel YA, Feng L, Perepelov AV, Senchenkova SN, Reeves PR, Wang L 2014 Structural diversity in Salmonella O antigens and its genetic basis. FEMS Microbiol Rev 38:56–89. doi:10.1111/1574-6976.12034. 23848592

[2] Geno KA, Gilbert GL, Song JY, Skovsted IC, Klugman KP, Jones C, Konradsen HB, Nahm MH 2015 Pneumococcal capsules and their types: past, present, and future. Clin Microbiol Rev 28:871–899. doi:10.1128/CMR.00024-15. 26085553 PMC4475641

[3] Dowell SF, Garman RL, Liu G, Levine OS, Yang YH 2001 Evaluation of Binax NOW, an assay for the detection of pneumococcal antigen in urine samples, performed among pediatric patients. Clin Infect Dis 32:824–825. doi:10.1086/319205. 11229853

[4] Bonten MJ, Huijts SM, Bolkenbaas M, Webber C, Patterson S, Gault S, van Werkhoven CH, van Deursen AM, Sanders EA, Verheij TJ, Patton M, McDonough A, Moradoghli-Haftvani A, Smith H, Mellelieu T, Pride MW, Crowther G, Schmoele-Thoma B, Scott DA, Jansen KU, Lobatto R, Oosterman B, Visser N, Caspers E, Smorenburg A, Emini EA, Gruber WC, Grobbee DE 2015 Polysaccharide conjugate vaccine against pneumococcal pneumonia in adults. N Engl J Med 372:1114–1125. doi:10.1056/NEJMoa1408544. 25785969

[5] Lovegrove A, Edwards CH, De Noni I, Patel H, El SN, Grassby T, Zielke C, Ulmius M, Nilsson L, Butterworth PJ, Ellis PR, Shewry PR 2017 Role of polysaccharides in food, digestion, and health. Crit Rev Food Sci Nutr 57:237–253. doi:10.1080/10408398.2014.939263. 25921546 PMC5152545

[6] Mohnen D 2008 Pectin structure and biosynthesis. Curr Opin Plant Biol 11:266–277. doi:10.1016/j.pbi.2008.03.006. 18486536

[7] Yu J, Lin J, Kim KH, Benjamin WH Jr, Nahm MH 2011 Development of an automated and multiplexed serotyping assay for *Streptococcus pneumoniae*. Clin Vaccine Immunol 18:1900–1907. doi:10.1128/CVI.05312-11. 21900529 PMC3209018

[8] Steffan W, Kovac P, Albersheim P, Darvill AG, Hahn MG 1995 Characterization of a monoclonal antibody that recognizes an arabinosylated (1–>6)-beta-D-galactan epitope in plant complex carbohydrates. Carbohydr Res 275:295–307. doi:10.1016/0008-6215(95)00174-R. 8529225

[9] Yang J, Nahm MH, Bush CA, Cisar JO 2011 Comparative structural and molecular characterization of *Streptococcus pneumoniae* capsular polysaccharide serogroup 10. J Biol Chem 286:35813–35822. doi:10.1074/jbc.M111.255422. 21859716 PMC3195564

[10] Goldblatt D, Plikaytis BD, Akkoyunlu M, Antonello J, Ashton L, Blake M, Burton R, Care R, Durant N, Feavers I, Fernsten P, Fievet F, Giardina P, Jansen K, Katz L, Kierstead L, Lee L, Lin J, Maisonneuve J, Nahm MH, Raab J, Romero-Steiner S, Rose C, Schmidt D, Stapleton J, Carlone GM 2011 Establishment of a new human pneumococcal standard reference serum, 007sp. Clin Vaccine Immunol 18:1728–1736. doi:10.1128/CVI.05252-11. 21852547 PMC3187044

[11] Burton RL, Nahm MH 2006 Development and validation of a fourfold multiplexed opsonization assay (MOPA4) for pneumococcal antibodies. Clin Vaccine Immunol 13:1004–1009. doi:10.1128/CVI.00112-06. 16960111 PMC1563573

[12] Harvey HA, Swords WE, Apicella MA 2001 The mimicry of human glycolipids and glycosphingolipids by the lipooligosaccharides of pathogenic neisseria and haemophilus. J Autoimmun 16:257–262. doi:10.1006/jaut.2000.0477. 11334490

[13] Yuki N 2005 Carbohydrate mimicry: a new paradigm of autoimmune diseases. Curr Opin Immunol 17:577–582. doi:10.1016/j.coi.2005.09.004. 16229995

[14] Heidelberger M, Rebers PA 1960 Immunochemistry of the pneumococcal types II, V, and VI. The relation of type VI to type II and other correlations between chemical constitution and precipitation in antisera to type VI. J Bacteriol 80:145–153. doi:10.1128/JB.80.2.145-153.1960. 14400599 PMC278834

[15] Felton LD, Prescott B, Kauffmann G, Ottinger B 1955 Antigens of vegetable origin active in pneumococcus infections. J Bacteriol 69:519–528. doi:10.1128/JB.69.5.519-528.1955. 14381369 PMC357578

[16] Spencer BL, Shenoy AT, Orihuela CJ, Nahm MH 2017 The pneumococcal serotype 15C capsule is partially O-acetylated and allows for limited evasion of 23-valent pneumococcal polysaccharide vaccine-elicited anti-serotype 15B antibodies. Clin Vaccine Immunol 24:e00099-17. doi:10.1128/CVI.00099-17. 28637806 PMC5583466

[17] Macher BA, Galili U 2008 The Galalpha1,3Galalpha1,4GlcNAc-R (alpha-Gal) epitope: a carbohydrate of unique evolution and clinical relevance. Biochim Biophys Acta 1780:75–88. doi:10.1016/j.bbagen.2007.11.003. 18047841 PMC2271034

[18] Galili U, Mandrell RE, Hamadeh RM, Shohet SB, Griffiss JM 1988 Interaction between human natural anti-alpha-galactosyl immunoglobulin G and bacteria of the human flora. Infect Immun 56:1730–1737. doi:10.1128/IAI.56.7.1730-1737.1988. 3290105 PMC259469

[19] Commins SP, Satinover SM, Hosen J, Mozena J, Borish L, Lewis BD, Woodfolk JA, Platts-Mills TA 2009 Delayed anaphylaxis, angioedema, or urticaria after consumption of red meat in patients with IgE antibodies specific for galactose-alpha-1,3-galactose. J Allergy Clin Immunol 123:426–433. doi:10.1016/j.jaci.2008.10.052. 19070355 PMC3324851

[20] Crispell G, Commins SP, Archer-Hartman SA, Choudhary S, Dharmarajan G, Azadi P, Karim S 2019 Discovery of alpha-Gal-containing antigens in North American tick species believed to induce red meat allergy. Front Immunol 10:1056. doi:10.3389/fimmu.2019.01056. 31156631 PMC6533943

[21] Byres E, Paton AW, Paton JC, Lofling JC, Smith DF, Wilce MC, Talbot UM, Chong DC, Yu H, Huang S, Chen X, Varki NM, Varki A, Rossjohn J, Beddoe T 2008 Incorporation of a non-human glycan mediates human susceptibility to a bacterial toxin. Nature 456:648–652. doi:10.1038/nature07428. 18971931 PMC2723748

[22] Paul A, Padler-Karavani V 2018 Evolution of sialic acids: implications in xenotransplant biology. Xenotransplantation 25:e12424. doi:10.1111/xen.12424. 29932472 PMC6756921

[23] Brouns F, Theuwissen E, Adam A, Bell M, Berger A, Mensink RP 2012 Cholesterol-lowering properties of different pectin types in mildly hyper-cholesterolemic men and women. Eur J Clin Nutr 66:591–599. doi:10.1038/ejcn.2011.208. 22190137

[24] Kamerling JP 2000 Pneumococcal polysaccharides: a chemical view, p 81–114. In Tomasz A (ed), *Streptococcus pneumoniae* molecular biology & mechanisms of disease. Mary Ann Liebert, Inc, Larchmont, NY.

[25] Haq TA, Mason HS, Clements JD, Arntzen CJ 1995 Oral immunization with a recombinant bacterial antigen produced in transgenic plants. Science 268:714–716. doi:10.1126/science.7732379. 7732379

[26] Zhu Q, Berzofsky JA 2013 Oral vaccines: directed safe passage to the front line of defense. Gut Microbes 4:246–252. doi:10.4161/gmic.24197. 23493163 PMC3669171

[27] Concha C, Canas R, Macuer J, Torres MJ, Herrada AA, Jamett F, Ibanez C 2017 Disease prevention: an opportunity to expand edible plant-based vaccines? Vaccines (Basel) 5:14. doi:10.3390/vaccines5020014. PMC5492011 28556800

[28] Commins SP 2015 Carbohydrates as allergens. Curr Allergy Asthma Rep 15:492. doi:10.1007/s11882-014-0492-y. 25430953

[29] Hamad A, Burks W 2017 Oral tolerance and allergy. Semin Immunol 30:28–35. doi:10.1016/j.smim.2017.07.001. 28739336

[30] Spiro MD, Kates KA, Koller AL, O'Neill MA, Albersheim P, Darvill AG 1993 Purification and characterization of biologically active 1,4-linked alpha-D-oligogalacturonides after partial digestion of polygalacturonic acid with endopolygalacturonase. Carbohydrate Res 247:9–20. doi:10.1016/0008-6215(93)84237-Z.

[31] York WS, Darvill AG, McNeil M, Stevenson TT, Albersheim P 1986 Isolation and characterization of plant cell walls and cell wall components. Methods Enzymol 118:3–40. doi:10.1016/0076-6879(86)18062-1.

[32] Park IH, Pritchard DG, Cartee R, Brandao A, Brandileone MC, Nahm MH 2007 Discovery of a new capsular serotype (6C) within serogroup 6 of *Streptococcus pneumoniae*. J Clin Microbiol 45:1225–1233. doi:10.1128/JCM.02199-06. 17267625 PMC1865839

[33] Geno KA, Saad JS, Nahm MH 2017 Discovery of novel pneumococcal serotype 35D, a natural WciG-deficient variant of serotype 35B. J Clin Microbiol 55:1416–1425. doi:10.1128/JCM.00054-17. 28202800 PMC5405259

[34] Sung CK, Li H, Claverys JP, Morrison DA 2001 An *rpsL* cassette, Janus, for gene replacement through negative selection in *Streptococcus pneumoniae*. Appl Environ Microbiol 67:5190–5196. doi:10.1128/AEM.67.11.5190-5196.2001. 11679344 PMC93289

[35] Goetz S, Rejzek M, Nepogodiev SA, Field RA 2016 The impact of aminopyrene trisulfonate (APTS) label in acceptor glycan substrates for profiling plant pectin beta-galactosyltransferase activities. Carbohydr Res 433:97–105. doi:10.1016/j.carres.2016.07.017. 27479753 PMC5036537

[36] Burton RL, Antonello J, Cooper D, Goldblatt D, Kim KH, Plikaytis BD, Roalfe L, Wauters D, Williams F, Xie GL, Nahm MH, Akkoyunlu M 2017 Assignment of opsonic values to pneumococcal reference serum 007sp for use in opsonophagocytic assays for 13 serotypes. Clin Vaccine Immunol 24:e00457-16. doi:10.1128/CVI.00457-16. 27974397 PMC5299120

