# Erratum for Nahm et al., “A Common Food Glycan, Pectin, Shares an Antigen with Streptococcus pneumoniae Capsule”

**DOI:** 10.1128/mSphere.00363-20

**Published:** 2020-04-29

**Authors:** Moon H. Nahm, Jigui Yu, Jiri Vlach, Maor Bar-Peled

**Affiliations:** a Department of Medicine, Division of Pulmonary, Allergy & Critical Care, University of Alabama at Birmingham, Birmingham, Alabama, USA; b Department of Microbiology, University of Alabama at Birmingham, Birmingham, Alabama, USA; c Complex Carbohydrate Research Center, University of Georgia, Athens, Georgia, USA

## ERRATUM

Volume 5, no. 2, e00074-20, 2020, https://doi.org/10.1128/mSphere.00074-20. In the 6th sentence of the abstract, “1,6-β-galactosidase” should read “terminal 1,6-linked β-galactose.”

Page 4 of the PDF, line 4: “1,6-β-galactosidase” should read “1,6-linked β-galactose.”

