# Supplementary figures and images for: A Common Food Glycan, Pectin, Shares an Antigen with Streptococcus pneumoniae Capsule
Source: mSphere. 2020 Apr 8;5(2):e00074-20. doi: 10.1128/mSphere.00074-20 (PMC7142292; doi:10.1128/mSphere.00074-20)

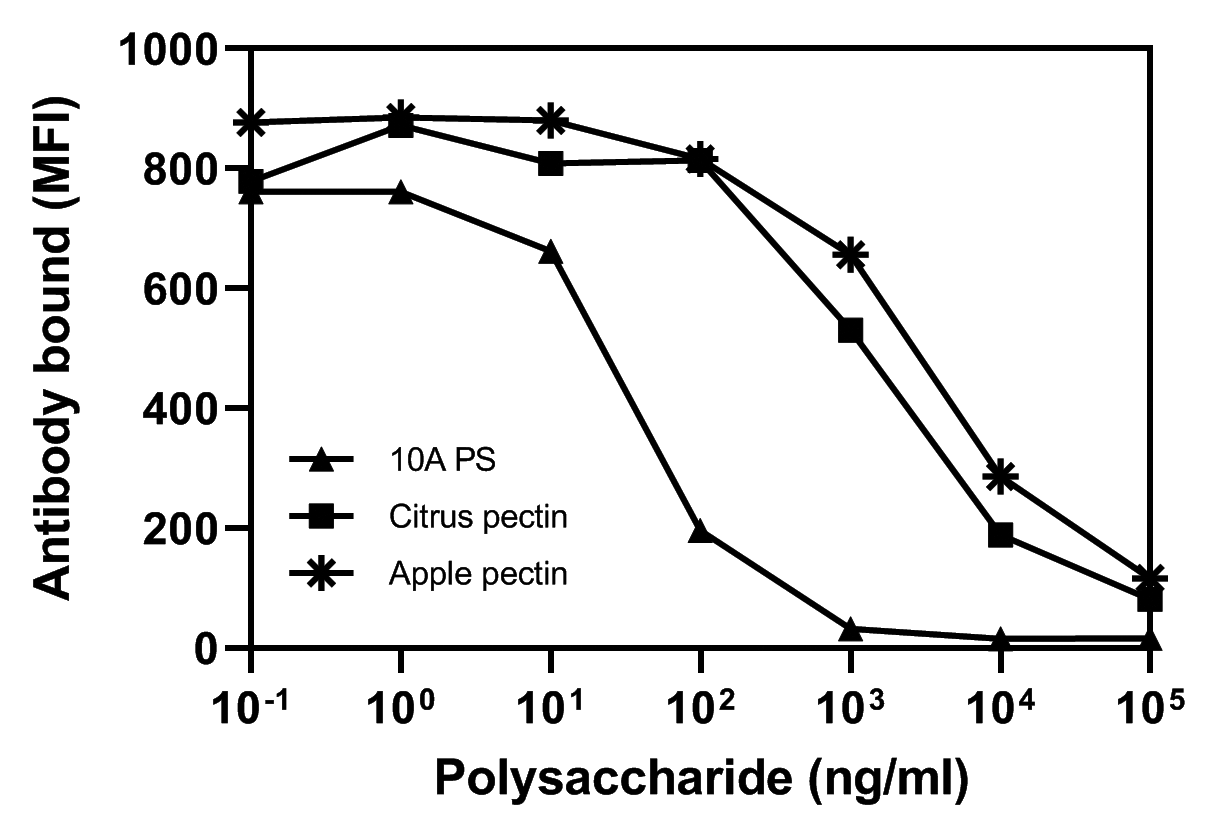

Supplement: FIG S1 [file mSphere.00074-20-sf001.tif]

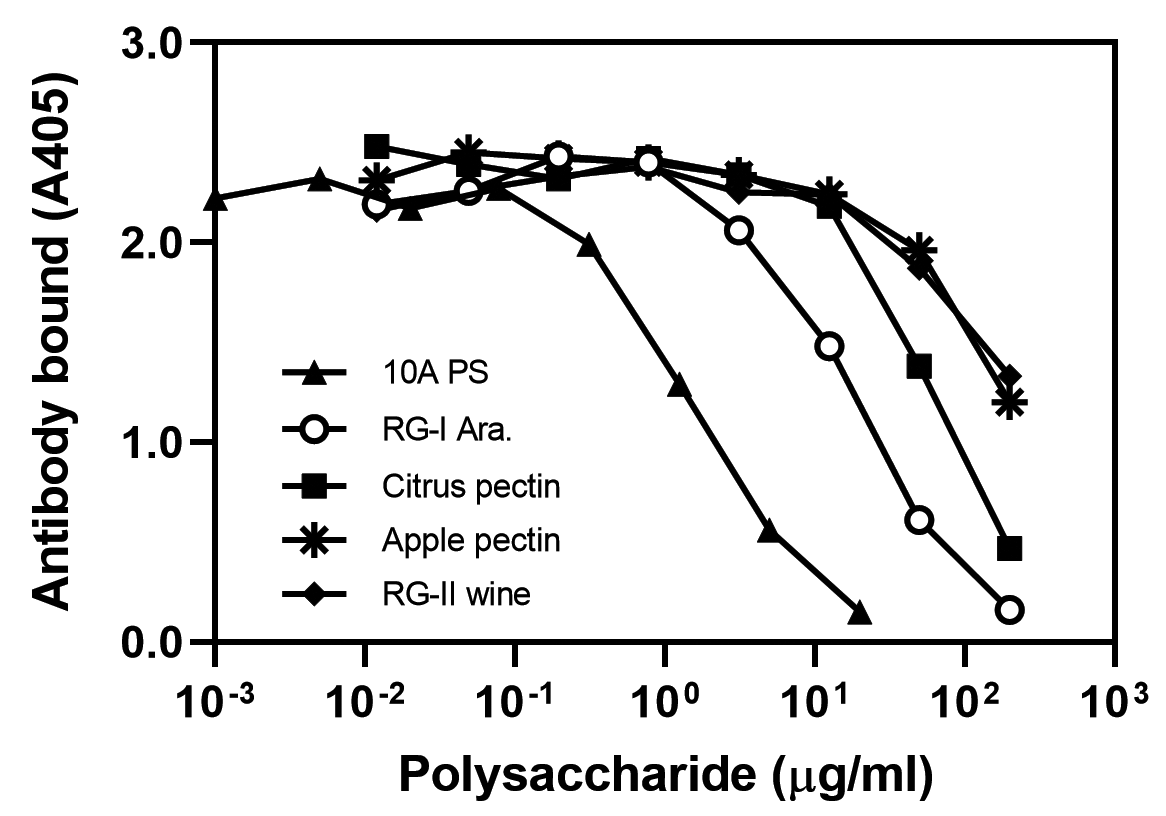

Supplement: FIG S2 [file mSphere.00074-20-sf002.tif]
